# P03-005 - MEFV heterozygous mutations in PFAPA patients

**DOI:** 10.1186/1546-0096-11-S1-A200

**Published:** 2013-11-08

**Authors:** A Kozlova, O Barabanova, N Kuzmenko, N Zinovieva, O Molochnikova, A Shcherbina

**Affiliations:** 1 Immunology, Federal Research and Clinical Center of Pediatric Hematology, Oncology, and Immunology, Moscow, Russian Federation; 2 Immunology, Children's Clinical Hospital N9, Moscow, Russian Federation

## Introduction

PFAPA syndrome (acronym for periodic fever, aphthous stomatitis, pharyngitis and cervical adenitis) is the most common cause of periodic fever in childhood. It is considered part of the wide family of the autoinflammatory diseases, but a genetic or molecular marker hasn't been identified yet, therefore, its etiology is still unknown. Diagnosis is essentially based on clinical criteria but, especially in younger children, it is sometimes difficult to differentiate it from other hereditary periodic fever syndromes. Familial (Mediterranean) fever (FMF) is caused by MEFV gene mutations, mostly inherited in autosomal recessive fashion. Yet, there are reports of heterozygous MEFV mutation carriers with clinical features of FMF.

## Objectives

To assess the effects of heterozygous MEFV mutations on the clinical features of PFAPA syndrome.

## Methods

We studied 27 patients with typical clinical manifestations of PFAPA syndrome, ages 2 -12 years (mean age 9.56 years). Direct sequencing of MEFV gene was performed, 21 patient had no mutations, and 6 patients had various heterozygous mutations of MEFV. We analyzed the spectrum of clinical features, response to prednisone treatment and tonsillectomy in patients who carried MEFV mutations in the heterozygous state and patients without mutations.

## Results

In comparison with PFAPA group without MEFV mutation patients with heterozygous mutations in MEFV gene had the following features: the symptoms begin at a somewhat younger age (15.7 months versus 19 months), the duration of attacks was shorter (3.92 days vs 5,1 days) and the interval between the attacks of the disease was found to be shorter, these patients rarely has had arthralgias, hepatosplenomegaly and aphthous stomatitis was present less frequently, during the attack CRP levels were higher, yet (unlike HPF) they always normalized between the attacks. In the group of carriers of heterozygous MEFV mutations all patients responded to steroid treatment and had full effect after tonsillectomy.

## Conclusion

In opposite to some other reports in our PFAPA group, patients with heterozygous MEFV mutation had typical PFAPA symptoms and no features suspicious of FMF. Thus, the pathologic meaning of the MEFV mutation in these group is not clear and does not seem to influence the course of the disease.

## Competing interests

None Declared.

